# Combined effects of constant temperature and radio frequency exposure on Aedes mosquito development

**DOI:** 10.1038/s41598-025-09383-3

**Published:** 2025-08-20

**Authors:** Nazri Che Dom, Rahmat Dapari, Nik Muhammad Hanif Nik Abdull Halim, Ahmad Taufek Abdul Rahman

**Affiliations:** 1 Faculty of Health Sciences, Universiti Teknologi MARA (UiTM), UITM Cawangan Selangor, Puncak Alam, 42300 Selangor Malaysia; 2 Integrated Mosquito Research Group (I-MeRGe), Universiti Teknologi MARA (UiTM), Puncak Alam, 42300 Selangor Malaysia; 3 Institute for Biodiversity and Sustainable Development (IBSD), Universiti Teknologi MARA, Shah Alam, Malaysia; 4 Faculty of Medicine and Health Sciences, Universiti Putra Malaysia, Serdang, 43400 Selangor Malaysia; 5 Integrated Dengue Research and Development, Faculty of Medicine and Health Sciences, Universiti Putra Malaysia, Serdang, 43400 Selangor Malaysia; 6 School of Physics and Material Studies, Faculty of Applied Sciences, Universiti Teknologi MARA, Shah Alam, 40450 Selangor Malaysia; 7 Centre for Astrophysics and Applied Radiation, Institute of Science, Universiti Teknologi MARA, Shah Alam, 40450 Selangor Malaysia

**Keywords:** *Ae. aegypti*, *Ae. albopictus*, Developmental stages, Radio frequency, Temperature, Vector control, Infectious diseases, Environmental sciences, Environmental impact, Public health

## Abstract

Mosquito-borne diseases, such as dengue, Zika, and chikungunya, pose significant public health threats, particularly in tropical regions like Malaysia. *Aedes aegypti* and *Aedes albopictus* are primary vectors of these diseases, with their developmental stages being highly sensitive to environmental factors. While temperature is a well-known driver of mosquito biology, the potential influence of anthropogenic factors such as radio frequency (RF) exposure remains underexplored. This study investigates the combined effects of temperature and RF exposure on the developmental stages of these mosquito species to provide insights into their population dynamics and inform vector control strategies. A factorial experimental design was employed, incorporating four temperature conditions (20 °C, 25 °C, 30 °C, and 35 °C) and three RF exposure levels (900 MHz, 18 GHz, and a control group with no RF exposure). The developmental durations for hatching, larval, pupation, and adult emergence stages were monitored daily under controlled laboratory conditions. Data were analyzed using a quadratic response surface model to evaluate the main effects and interactions between temperature and RF exposure. Temperature emerged as the dominant factor influencing developmental durations, with optimal conditions observed at 30–32 °C. RF exposure, particularly at 18 GHz, acted as a secondary modulating factor, accelerating developmental stages under certain temperature conditions. *Ae. aegypti* exhibited greater sensitivity to temperature changes compared to *Ae. albopictus*, which displayed higher adaptability and resilience to environmental variations. Interaction effects were most evident at intermediate temperatures (25–30 °C), where RF exposure synergistically reduced developmental durations. However, extreme RF exposure levels and suboptimal temperatures prolonged developmental periods. This study highlights the critical role of temperature in mosquito development while identifying RF exposure as a potential modulator under specific conditions. The findings underscore the importance of considering both environmental and anthropogenic factors in vector management strategies. Future research should explore the molecular mechanisms underlying these interactions to refine predictive models and enhance vector control efforts in rapidly urbanizing regions.

## Introduction

Understanding how environmental variables such as temperature and anthropogenic stressors interact is essential for anticipating mosquito population dynamics and strengthening vector control strategies. As ectothermic organisms, mosquitoes are highly sensitive to external environmental conditions. Temperature, in particular, plays a critical role in regulating their developmental rates, survival, and reproductive potential. Beyond traditional ecological drivers, there is growing concern about the biological impacts of radiofrequency (RF) electromagnetic fields, especially as exposure intensifies with expanding telecommunication infrastructure. While temperature has long been recognized as a key determinant in mosquito development, RF exposure remains a less-explored variable despite increasing global prevalence. Initial findings suggest that RF may influence insect physiology and development through non-thermal mechanisms, including oxidative stress and calcium ion channel disruption, which could subsequently alter behavior and vector competence^1–3^. Integrating RF into predictive ecological models offers the potential to improve the spatial and temporal precision of vector risk maps and inform targeted interventions. This is particularly relevant in the context of integrated vector management (IVM), which promotes tailored, evidence-based control measures that consider both environmental and anthropogenic factors^4–6^.

Despite a few studies evaluating temperature, humidity, and light effects on mosquito development^7–10^, research examining how RF interacts with these natural variables remains sparse. Given the pervasiveness of RF-emitting devices such as mobile phones, Wi-Fi routers, and satellite systems in densely populated urban areas, the potential for RF to influence insect life history traits warrants more focused investigation^11–15^. This is especially urgent in Malaysia, where high dengue incidence coexists with intense urban development and expanding RF infrastructure. These unique environmental conditions may synergistically influence *Aedes aegypti* and *Aedes albopictus*, the principal vectors of dengue and other arboviruses^16^.

This study investigates the combined effects of temperature and RF exposure on key developmental stages of these two mosquito species. We aim to identify environmental conditions both optimal and suboptimal that shape hatching, larval, pupation, and adult emergence periods. The findings will provide insight into how concurrent ecological and anthropogenic variables influence mosquito population dynamics, contributing to the design of more effective and locally relevant vector control strategies.

## Methods

### Study design

This study was designed to evaluate the combined effects of temperature and radio frequency (RF) exposure on the developmental stages of *Ae. aegypti* and *Ae. albopictus*. A factorial experimental approach was employed, integrating four temperature conditions (20 °C, 25 °C, 30 °C, and 35 °C) with three RF exposure levels (900 MHz, 18 GHz, and a control group with no RF exposure). The design aimed to capture both the independent effects of temperature and RF exposure, as well as their potential interaction on mosquito development, including hatching, larval, pupation, and adult emergence periods.

Experiments were conducted under controlled laboratory conditions to minimize external variability. Environmental chambers with precise temperature regulation were used to simulate the selected temperature treatments. RF exposure was generated using calibrated RF sources capable of producing 900 MHz and 18 GHz frequencies, ensuring uniform exposure across the experimental setup. The control group was maintained under identical conditions without RF exposure to serve as a baseline for comparison. To achieve a robust and unbiased assessment, each experimental condition was replicated three times, with a minimum of 50 individuals per replicate for each mosquito species. This replication ensured adequate statistical power and accounted for biological variability. In total, 1,800 larvae of *Ae. aegypti* and 1,800 larvae of *Ae. albopictus* were used in this study, corresponding to 12 experimental groups × 3 replicates × 50 larvae per replicate per species. The experimental groups were organized into a total of 12 combinations, representing all possible pairings of the four temperature levels and three RF exposure levels. Mosquitoes were randomly assigned to these groups to prevent systematic bias.

The developmental stages of the mosquitoes were monitored daily, with specific attention to the time required for transitions between life stages (hatching, larval development, pupation, and adult emergence). These durations were recorded in days to provide quantitative data for subsequent analysis. The experimental design allowed for the evaluation of both linear and non-linear effects of temperature and RF exposure, as well as their interaction effects, using response surface methodology. This comprehensive study design ensures that the results provide a detailed understanding of how environmental factors influence mosquito development, offering valuable insights for potential applications in vector control strategies.

### Mosquito rearing

Laboratory-reared colonies of *Ae. aegypti* and *Ae. albopictus* were used for this study. Mosquitoes were maintained under controlled conditions at 27 ± 2 °C, 80 ± 10% relative humidity, and a 12:12-hour light-dark photoperiod. Adult mosquitoes were housed in mesh cages (30 × 30 × 30 cm) and provided with a 10% sucrose solution ad libitum. Females were blood-fed using a membrane feeding system to stimulate oviposition, following ethical guidelines approved by the institutional review board. Eggs were collected on oviposition papers, dried, and stored at 25 °C in humidified containers. For experimental purposes, eggs were hatched in dechlorinated tap water. Larvae were reared in plastic trays containing 1 L of water and fed daily with a 3:1 mixture of finely ground fish food (TetraMin^®^) and yeast powder. Water was replaced every two days to maintain quality. Pupae were collected manually and placed in fresh water until adult emergence. A synchronized cohort of 50 eggs from each species was allocated to each experimental group, ensuring uniformity across treatments. This standardized protocol minimized variability and provided a consistent basis for assessing the effects of temperature and RF exposure on mosquito developmental stages^17^.

### Experimental setup

To evaluate the effects of temperature on mosquito development, four temperature conditions (20 °C, 25 °C, 30 °C, and 35 °C) were established. These temperatures were selected to represent a range of environmental conditions that mosquitoes might encounter in natural settings. Temperature treatments were maintained using humidified incubators (Model: BMT Friocell 404 (C111082). The incubators were calibrated to ensure precise temperature control (± 0.5 °C) and were equipped with humidity regulators to maintain consistent relative humidity (80 ± 10%). Mosquito trays for each experimental group were randomly placed within the incubators to minimize positional bias. A 12:12-hour light-dark photoperiod was maintained throughout the experiment to simulate natural light cycles.

Three RF exposure conditions were included: 900 MHz, 18 GHz, and a control group with no RF exposure. These frequencies were selected based on their relevance to modern telecommunication systems (900 MHz for cellular networks and 18 GHz for higher-frequency applications). RF exposure was generated using a custom-built polystyrene exposure chamber (49.5 cm × 62.0 cm × 37.0 cm), equipped with a wideband antenna (A-Info LB8180-NF; 0.8–18 GHz, 12 dB gain). The RF source was a wideband signal generator (Keysight N5173B EXG X-Series; 9 kHz–40 GHz) connected to a super ultra-wideband amplifier. The control group was placed in a separate incubator with identical environmental conditions but without RF exposure to avoid unintentional interference from the RF signal. Exposure duration for all treatments was set to 24 h per day for the entire developmental period of the mosquitoes, ensuring continuous exposure during the experiment^18^.

To achieve the unique combinations of temperature and RF exposure, RF exposure chambers were placed directly inside each incubator that had been pre-set to one of the target temperature conditions (20 °C, 25 °C, 30 °C, or 35 °C). This configuration enabled simultaneous and continuous exposure of mosquito trays to a specific RF frequency (900 MHz or 18 GHz) while maintaining strict temperature control. Each RF exposure condition was matched with each temperature level, resulting in 12 unique treatment groups, including control groups. This physical setup allowed for the systematic assessment of both independent and interactive effects of temperature and RF on mosquito development, in line with the factorial experimental design.

### Developmental stage monitoring

The developmental progress of *Ae. aegypti* and *Ae. albopictus* was monitored daily to evaluate the effects of temperature and radio frequency (RF) exposure on four key life stages: hatching, larval, pupation, and adult emergence periods. The hatching period was defined as the time from the submersion of eggs in dechlorinated tap water to the emergence of first-instar larvae. Eggs were submerged in labeled plastic trays under the assigned experimental conditions and inspected every 12 h using a stereomicroscope. The time of first larval emergence was recorded, and the hatching period was calculated as the difference between the time of egg submersion and larval emergence.

The larval period was measured as the time from first-instar larval emergence to the initiation of pupation. Larvae were maintained in trays containing 1 L of dechlorinated water and fed daily with a finely ground diet. Trays were inspected every 12 h, and pupae were manually separated from larvae using a fine mesh strainer. The larval period was recorded as the duration between the emergence of first-instar larvae and the observation of the first pupa. The pupation period was defined as the time from pupal formation to the emergence of adult mosquitoes. Pupae were transferred to small cups containing fresh dechlorinated water and placed in mesh cages under the respective experimental conditions. Monitoring was conducted every 12 h to record the time of adult emergence. Similarly, the adult emergence period was defined as the duration from pupal formation to the complete emergence of adult mosquitoes, and any incomplete emergence events were excluded from the analysis.

To account for variability in development time, all developmental durations were recorded at the individual level across all replicates within each experimental group. This allowed us to capture both inter-individual and inter-replicate variation. Statistical analysis (ANOVA) was conducted using mean values per replicate, while standard deviation and standard error were calculated to reflect variability within groups. This approach ensured a robust interpretation of differences between treatment conditions.

All experimental groups were inspected twice daily to ensure no developmental transitions were missed. Observations were conducted using a stereomicroscope for accuracy, and all data were systematically recorded in structured datasheets, including the date and time of each transition. This comprehensive monitoring protocol ensured accurate and reproducible measurements of developmental stages, providing robust data for evaluating the influence of temperature and RF exposure on mosquito life cycles.

### Data analysis

The data collected from the developmental stages of *Ae. aegypti* and *Ae. albopictus* were analyzed using analysis of variance (ANOVA) to evaluate the effects of radio frequency (RF) exposure and temperature on hatching, larval, pupation, and adult emergence periods. A quadratic model for response surface analysis was employed to quantify the main effects of RF exposure and temperature, as well as their interaction effects (RF × temperature), on mosquito development. The statistical significance of the interaction terms was assessed to determine whether RF exposure and temperature jointly influenced the developmental parameters.

Prior to performing ANOVA, data were evaluated to ensure compliance with the assumptions of parametric testing. The Shapiro–Wilk test was used to assess the normality of the data distributions for each experimental group, while homogeneity of variances was verified using Levene’s test. Both tests confirmed that the assumptions of normality and equal variances were satisfied (*p* > 0.05 for all comparisons). Independence of observations was ensured through random assignment of mosquito larvae and the use of separate biological replicates for each group.

Model validation was conducted to ensure the robustness and reliability of the statistical findings. Goodness-of-fit measures, including F-values and p-values, were used to evaluate the adequacy of the models in explaining the variability in the developmental data. Post-hoc tests, such as Tukey’s Honest Significant Difference (HSD) test, were performed to facilitate pairwise comparisons between treatment groups and identify specific differences in developmental outcomes across temperature and RF exposure levels.

All statistical analyses were performed using IBM SPSS Statistics (v25). The software was used for both ANOVA and post-hoc testing, as well as for generating response surface models and interaction plots to visualize the effects of the experimental factors on mosquito development. This comprehensive approach ensured the accuracy and reproducibility of the statistical analysis, providing meaningful insights into the influence of environmental factors on mosquito life cycles.

### Ethics statement

This study was conducted in compliance with ethical standards and guidelines for animal research. The research protocol was reviewed and approved by the Committee on Animal Research and Ethics (UiTM CARE) under the reference number UiTM CARE: 377/2022, dated 13th May 2022. The approved duration for the study was from July 2022 to June 2023. All experimental procedures involving animals were carried out with strict adherence to institutional and national ethical guidelines to ensure the humane treatment of the animals involved.

## Results

### Effect of temperature and radio frequency (RF) exposure on the developmental stages of *Ae. aegypti* and *Ae. albopictus*

**Fig. 1:**
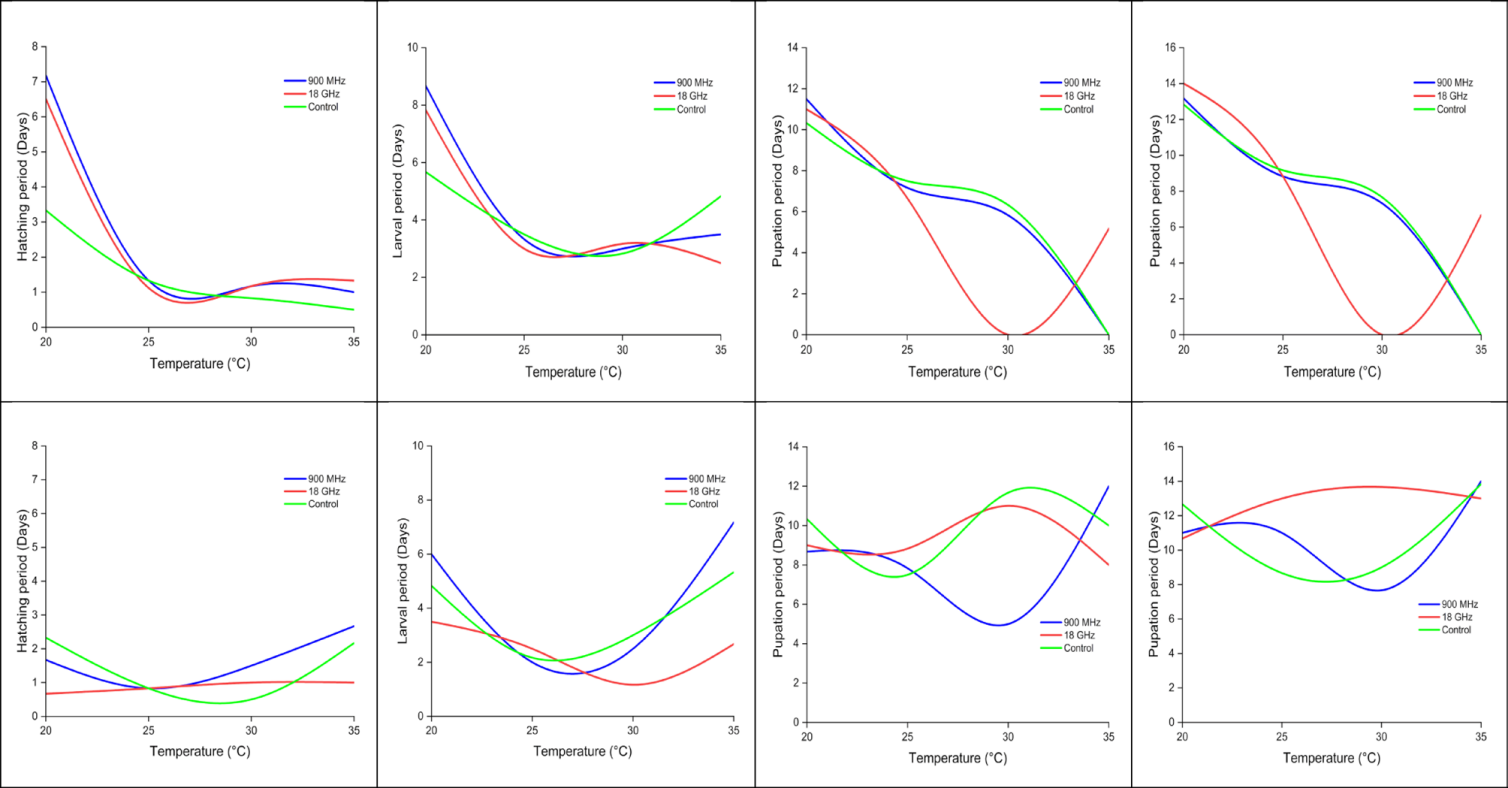
Effect of temperature (°C) and radio frequency (RF) exposure (900 MHz and 18 GHz) on the developmental stages of *Ae. aegypti* (upper panels) and *Ae. albopictus* (lower panels), including hatching, larval, and pupation periods (days). The developmental durations are compared across three conditions: 900 MHz, 18 GHz, and control (no RF exposure).

Figure 1 illustrates the effects of temperature and radio frequency (RF) exposure (900 MHz and 18 GHz) on the developmental stages of *Ae. aegypti* and *Ae. albopictus*. Across all developmental stages, temperature significantly influences the duration of each period, with RF exposure modulating these effects to varying degrees. For both species, the hatching period decreases as temperature increases from 20 °C to approximately 30 °C, after which it stabilizes or slightly increases at 35 °C. Notably, *Ae. albopictus* demonstrates a sharper decline in hatching duration compared to *Ae. aegypti*, suggesting a greater adaptability to rising temperatures. RF exposure, particularly at 18 GHz, consistently reduces hatching durations compared to the control group, with the effect being most prominent at lower temperatures.

The larval period also shows a consistent decline with increasing temperatures for both species, with a plateau or marginal increase at 35 °C. *Ae. albopictus* exhibits a steeper reduction in the larval period, achieving shorter durations at intermediate and higher temperatures (30 °C and 35 °C) compared to *Ae. aegypti*. RF exposure further accelerates larval development, with 18 GHz having the most pronounced effect, particularly at 25 °C and 30 °C. The control group generally displays the longest larval durations across all temperatures, highlighting the influence of RF exposure on larval development. In the pupation period, a non-linear trend is observed, with durations decreasing at intermediate temperatures (25 °C to 30 °C) but stabilizing or slightly increasing at 35 °C. While *Ae. albopictus* consistently shows shorter pupation durations than *Ae. aegypti*, the variability across RF exposure conditions is more pronounced in *Ae. aegypti*. RF exposure, especially at 18 GHz, significantly reduces the pupation period at lower temperatures but has diminishing effects as temperature increases. At higher temperatures (e.g., 35 °C), the pupation durations under RF exposure and control conditions converge, suggesting a temperature-dominant effect at this extreme.

The interaction between temperature and RF exposure is most apparent at lower and intermediate temperatures (20 °C to 30 °C), where RF exposure, particularly at 18 GHz, synergistically reduces the developmental durations of both species. However, at higher temperatures (35 °C), the impact of RF exposure diminishes, with temperature alone dictating developmental outcomes. Across all conditions, *Ae. albopictus* completes its developmental stages faster than *Ae. aegypti*, indicating a species-specific sensitivity to environmental variables. The findings suggest that RF exposure, particularly at 18 GHz, accelerates the developmental progression of both species, with *Ae. albopictus* being more responsive to these conditions. This interaction between temperature and RF exposure provides valuable insights into the potential influence of environmental and anthropogenic factors on mosquito development, which could inform strategies for vector control.

### Influence of temperature and RF exposure on developmental stages of Aedes mosquitoes: species-specific sensitivities and trends

The results presented in Table 1 demonstrate the influence of temperature and radio frequency (RF) exposure on the developmental stages of *Ae. aegypti* and *Ae. albopictus*. For *Ae. aegypti*, temperature emerged as the dominant factor across multiple developmental stages, with significant effects observed for the hatching (*p* = 0.01), larval (*p* = 0.01), and pupation (*p* = 0.01) periods. The quadratic effect of temperature (B²) was particularly important in shaping the non-linear responses observed in these stages, consistent with prior findings that temperature plays a critical role in accelerating mosquito development within optimal ranges. Interestingly, while RF exposure (A) alone did not significantly affect hatching, larval, or pupation durations (*p* > 0.05), the 18 GHz RF exposure identified in previous studies as potentially modulating mosquito physiology appears to contribute indirectly, as indicated by trends in shorter durations under RF-exposed conditions. However, these effects were not statistically significant, highlighting the predominant role of temperature in shaping developmental outcomes.

In contrast, *Ae. albopictus* exhibited fewer significant responses to temperature and RF exposure. The quadratic effect of temperature (B²) significantly influenced the larval period (*p* = 0.01), aligning with previous findings that *Ae. albopictus* is well-adapted to temperature fluctuations, particularly in warmer environments. However, temperature and RF exposure had minimal impact on the hatching and pupation periods (*p* > 0.05), suggesting a species-specific resilience or lower sensitivity to environmental variables compared to *Ae. aegypti*. These findings corroborate earlier observations that *Ae. albopictus* tends to exhibit faster developmental rates and greater adaptability to varying conditions, particularly at higher temperatures.

Neither species showed significant effects of RF exposure on the adult emergence period (*p* > 0.05), indicating that temperature, rather than RF exposure, plays a more critical role in determining overall developmental success. The lack of significant interaction effects (AB) across most stages further supports the conclusion that temperature acts as the primary driver of developmental changes, while RF exposure appears to have a minor or non-linear influence that requires further investigation. These findings align with prior studies demonstrating that temperature within optimal ranges accelerates mosquito development, while extreme temperatures can lead to prolonged or suboptimal development. Additionally, the current results reinforce the limited direct impact of RF exposure on mosquito development, suggesting that the observed trends in shorter durations under 18 GHz RF exposure may be more prominent under specific temperature conditions.

**Table 1:** Analysis of variance (ANOVA) of the quadratic model for response surface on the effects of radio frequency (RF) exposure (900 MHz and 18 GHz) and temperature (°C) on the hatching, larval, pupation & emergence periods of *Ae. aegypti* and *Ae. albopictus*.

Sources	*Ae. aegypti*					*Ae. albopictus*				
	Sum of squares	df	Mean square	*F*-value	*p*-value	Sum of squares	df	Mean square	*F*-value	*p*-value
I. Hatching period										
*Model*	48.23	5	9.65	7.24	0.02**	3.72	5	0.74	2.21	0.18
*A* - RF exposure	2.35	1	2.35	1.77	0.23	0.68	1	0.68	2.02	0.21
*B* - Temperature	29.41	1	29.41	22.07	0.01	0.24	1	0.24	0.71	0.43
AB	0.18	1	0.17	0.13	0.73	0.01	1	0.01	0.01	0.97
*A*²	2.37	1	2.37	1.78	0.23	0.12	1	0.12	0.36	0.57
*B*²	13.82	1	13.82	10.38	0.02	2.10	1	2.10	6.24	0.05
** Residual**	7.99	6	1.33			2.02	6	0.34		
** Cor total**	56.22	11				5.74	11			
**II. Larval period**										
*Model*	39.08	5	7.82	6.79	0.02**	31.54	5	6.31	6.73	0.02**
*A* - RF exposure	0.01	1	0.01	0.01	0.92	3.77	1	3.77	4.02	0.09
*B* - Temperature	21.73	1	21.73	18.88	0.01	0.01	1	0.01	0.01	0.934
AB	1.53	1	1.53	1.33	0.29	1.60	1	1.60	1.70	0.24
*A*²	0.37	1	0.37	0.32	0.59	0.90	1	0.90	0.96	0.37
*B*²	16.73	1	16.73	14.54	0.01	21.76	1	21.76	23.22	0.01
** Residual**	6.91	6	1.15			5.62	6	0.94		
** Cor total**	45.99	11				37.16	11			
**III. Pupation period**										
*Model*	146.76	5	29.35	4.39	0.04**	10.34	5	2.07	0.38	0.85
*A* - RF exposure	0.22	1	0.22	0.03	0.86	0.89	1	0.89	0.16	0.70
*B* - Temperature	115.85	1	115.85	17.33	0.01	0.70	1	0.70	0.13	0.73
AB	3.13	1	3.13	0.47	0.52	1.14	1	1.14	0.21	0.67
*A*²	0.02	1	0.02	0.01	0.96	4.52	1	4.52	0.82	0.40
*B*²	1.69	1	1.69	0.25	0.63	3.17	1	3.17	0.58	0.48
** Residual**	40.11	6	6.69			33.04	6	5.51		
** Cor total**	186.87	11				43.38	11			
**IV. Adult Emergence period**										
*Model*	12395.01	5	2479.00	2.92	0.11	3016.50	5	603.30	1.10	0.45
*A* - RF exposure	2224.45	1	2224.45	2.62	0.16	1252.75	1	1252.75	2.28	0.18
*B* - Temperature	3110.99	1	3110.99	3.66	0.10	734.62	1	734.62	1.33	0.29
AB	2368.84	1	2368.84	2.79	0.15	647.48	1	647.48	1.18	0.32
*A*²	52.81	1	52.81	0.06	0.81	760.09	1	760.09	1.38	0.28
*B*²	1177.31	1	1177.31	1.39	0.28	547.02	1	547.02	0.99	0.36
** Residual**	5096.26	6	849.38			3303.94	6	550.66		
** Cor total**	17491.27	11				6320.44	11			

Note: Model A evaluates the effects of radio frequency (RF) exposure (900 MHz, 18 GHz, and control) on the developmental periods of *Ae. aegypti* and *Ae. albopictus*, including the main effects of RF exposure and its interaction with temperature. Model B assesses the effects of temperature (20 °C, 25 °C, 30 °C, and 35 °C) on the developmental periods, including the main effects of temperature and its interaction with RF exposure. The interaction effect (AB) represents the combined influence of RF exposure and temperature on the developmental outcomes.

### Temperature and radio frequency interaction effects on Aedes mosquito developmental durations

**Fig. 2:**
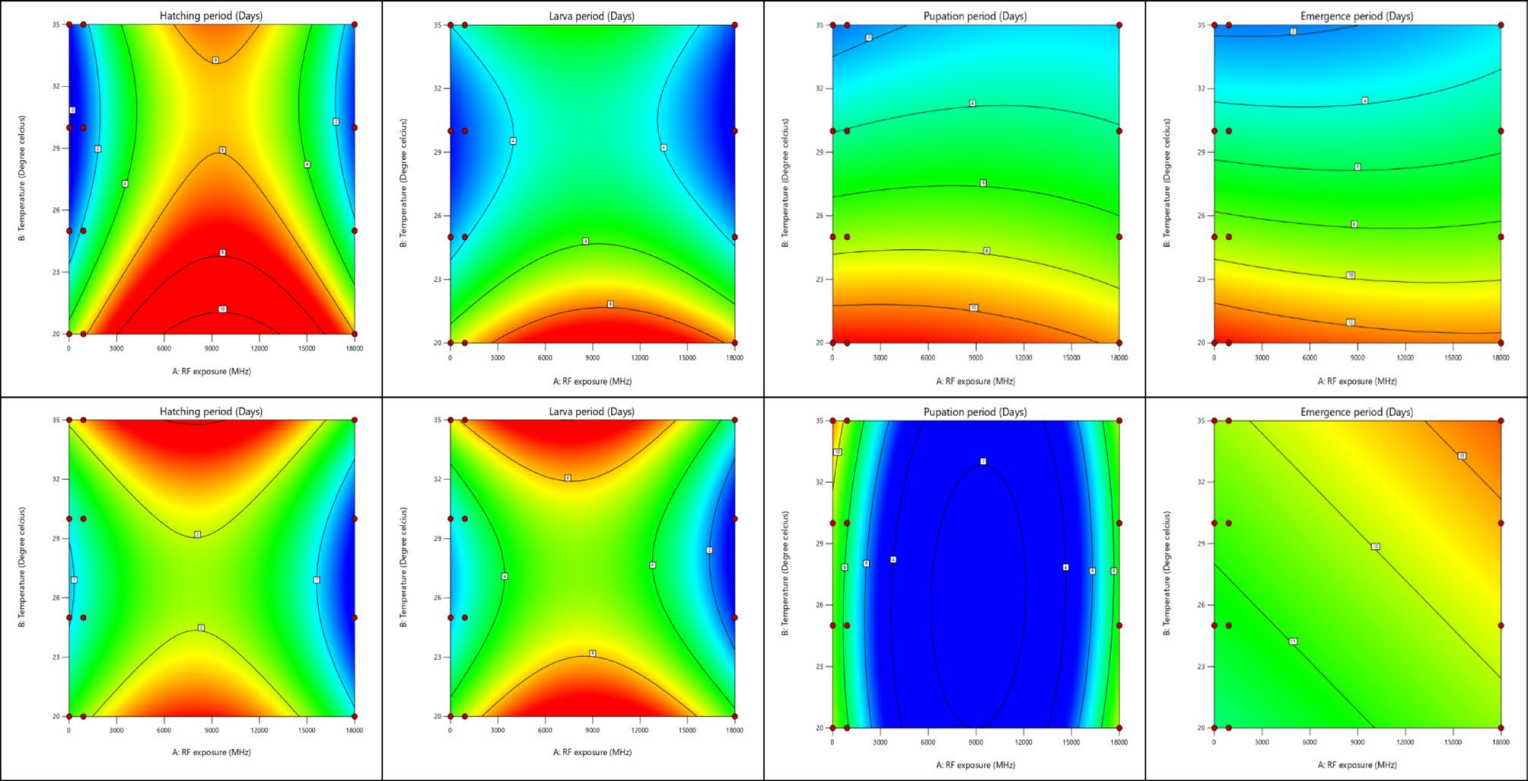
Interaction effects of RF exposure (MHz) and temperature (°C) on the developmental stages of *Ae. aegypti* (upper panels) and *Ae. albopictus* (lower panels), including hatching, larval, pupation, and emergence periods (days). The contour plots indicate the duration of each developmental stage under varying environmental conditions. Shorter durations are represented by blue-green gradients, while longer durations are indicated by red gradients. Optimal development occurs at mid-range RF exposure (~ 9,000 MHz) and higher temperatures (~ 30–32 °C), whereas lower temperatures and extreme RF exposure levels prolong developmental stages.

The contour plots in Fig. 2 depict the interaction effects of radio frequency (RF) exposure (MHz) and temperature (°C) on the developmental stages of *Ae. aegypti* (upper panels) and *Ae. albopictus* (lower panels), including hatching, larval, pupation, and emergence periods. The gradients, ranging from blue-green (shorter durations) to red (longer durations), illustrate how varying environmental conditions influence developmental timelines. Across all stages, optimal development for both species is observed at mid-range RF exposure levels (~ 9,000 MHz) combined with higher temperatures (~ 30–32 °C). In contrast, lower temperatures (< 25 °C) and extreme RF exposure levels (< 3,000 MHz or > 15,000 MHz) prolong developmental durations, as indicated by the red gradients.

For the hatching period, both species exhibit reduced durations at higher temperatures (25–32 °C), with a sharper decline observed for *Ae. albopictus*, reflecting its greater adaptability to temperature increases. RF exposure at mid-range levels (~ 9,000 MHz) further shortens hatching durations compared to lower or higher RF exposure levels. This pattern aligns with prior findings that temperature plays a dominant role in accelerating hatching, while RF exposure has a secondary modulating effect. The larval period also decreases significantly at higher temperatures (~ 30 °C) for both species, with the shortest durations occurring at mid-range RF exposure (~ 9,000 MHz). Extreme RF exposure levels or lower temperatures result in prolonged larval stages, consistent with earlier observations that suboptimal conditions delay development. *Ae. albopictus* demonstrates a more uniform response to temperature and RF exposure compared to *Ae. aegypti*, suggesting greater resilience to environmental fluctuations.

The pupation period reveals a non-linear trend, with the shortest durations observed at moderate RF exposure (~ 9,000 MHz) and temperatures between 30 °C and 32 °C. Lower temperatures and extreme RF exposure levels significantly prolong pupation, as shown by the red gradients. *Ae. aegypti* appears more sensitive to environmental variability, exhibiting a broader range of pupation durations compared to *Ae. albopictus*, which maintains relatively consistent pupation durations under similar conditions. For the emergence period, both species achieve the shortest durations under optimal conditions of higher temperatures (~ 30–32 °C) and mid-range RF exposure (~ 9,000 MHz). Prolonged emergence periods are evident at lower temperatures and extreme RF exposure levels. Unlike earlier stages, the emergence period shows less pronounced differences between the two species, reflecting the dominant influence of temperature at this stage rather than RF exposure.

Overall, the results underscore the critical role of temperature in driving mosquito developmental stages, with RF exposure acting as a secondary factor that modulates these effects. The optimal conditions of mid-range RF exposure and higher temperatures (~ 30–32 °C) facilitate faster development, while extreme RF levels and lower temperatures delay progression. *Aedes albopictus* consistently demonstrates greater adaptability to environmental variations compared to *Aedes aegypti*. These findings provide valuable insights into the combined influence of environmental and anthropogenic factors on mosquito development, which could inform targeted strategies for vector control under varying climatic conditions.

## Discussion

This study investigated the combined effects of temperature and radio frequency (RF) exposure on the developmental stages of *Ae. aegypti* and *Ae. albopictus*, revealing critical insights into their environmental sensitivities and adaptive capacities. The results demonstrate that temperature plays a dominant role in modulating mosquito developmental durations across all life stages, with RF exposure acting as a secondary modulating factor under specific conditions. These findings provide valuable contributions to our understanding of mosquito development under varying environmental conditions, with implications for vector control strategies.

The hatching period was significantly influenced by temperature in *Ae. aegypti* (*p* = 0.01), where increasing temperature shortened the duration, peaking at 30–32 °C for optimal development. RF exposure, particularly at mid-range levels (~ 9,000 MHz), further reduced the hatching period, though the effect was not statistically significant. Conversely, *Ae. albopictus* demonstrated less sensitivity to these factors, with only the quadratic effect of temperature approaching significance (*p* = 0.05). This aligns with previous findings that *Ae. albopictus* exhibits greater resilience to environmental stressors, potentially explaining its broader geographic distribution compared to *Ae. aegypti*^19^. The larval period showed consistent reductions at higher temperatures for both species, with significant quadratic effects of temperature for *Ae. aegypti* (*p* = 0.01) and *Ae. albopictus* (*p* = 0.01). Optimal conditions were observed at 30 °C combined with mid-range RF exposure (~ 9,000 MHz). Interestingly, RF exposure exhibited a greater influence on *Ae. albopictus* compared to *Ae. aegypti*, as indicated by trends in reduced larval durations. These results corroborate earlier studies highlighting the role of temperature as a primary driver of larval development while suggesting that RF exposure may interact with temperature to enhance development under specific conditions^20,21^.

The pupation period revealed a non-linear relationship with temperature for *Ae. aegypti* (*p* = 0.01), where durations were shortest at 30 °C and mid-range RF exposure. However, for *Ae. albopictus*, neither temperature nor RF exposure significantly affected the pupation period, indicating a lower dependence on environmental conditions during this stage. This species-specific difference emphasizes the adaptability of *Ae. albopictus* and its ability to maintain consistent development under a wider range of conditions. These findings align with previous observations that *Ae. aegypti* is more sensitive to environmental fluctuations, which may limit its ability to thrive in suboptimal conditions. This greater adaptability of *Ae. albopictus* to environmental extremes likely contributes to its broader geographic distribution, as supported by ecological studies emphasizing its resilience to fluctuating conditions^22–24^. For the adult emergence period, neither temperature nor RF exposure showed significant effects for either species, indicating that earlier developmental stages are more influenced by these factors. This finding is consistent with studies suggesting that temperature primarily affects the early life stages of mosquitoes, with diminishing influence as they progress toward adulthood^24–26^.

The interaction effects of temperature and RF exposure were most evident at intermediate temperatures (25–30 °C) and mid-range RF exposure levels (~ 9,000 MHz), where both species exhibited accelerated development. Extreme conditions, such as lower temperatures (< 25 °C) and high RF exposure (> 15,000 MHz), prolonged developmental durations, highlighting the adverse effects of environmental extremes. These results reinforce the importance of considering combined environmental factors in understanding mosquito development, as single-variable studies may overlook critical interactions.

Building upon previous research, studies have shown that environmental stressors such as temperature and RF exposure interact to influence biological processes^27^. The observed reduction in hatching periods under optimal temperature and RF exposure conditions aligns with studies that suggest RF exposure may interact with physiological mechanisms to accelerate developmental rates. This supports the hypothesis that RF exposure, while secondary to temperature, can act as a modulating factor under certain conditions. The reasoning behind these findings lies in the metabolic and physiological responses of mosquitoes to temperature. Optimal temperatures (30–32 °C) enhance enzymatic activity and energy utilization, aligning with existing ecological theories that suggest temperature drives key life-history traits. Similarly, while RF exposure may not directly influence hatching, it could act synergistically with temperature by inducing subtle biological responses that require further investigation. Backing these findings, studies have documented that *Ae. aegypti* is more sensitive to environmental variations compared to *Ae. albopictus*, which demonstrates greater resilience to stressors. For *Ae. albopictus*, the quadratic effect of temperature (*p* = 0.05) approached significance, suggesting non-linear relationships with hatching duration.

While temperature remains the dominant factor influencing the hatching period, the conditions under which RF exposure impacts developmental rates depend on factors such as intensity and frequency of exposure. For example, extreme RF exposure levels may act as stressors rather than facilitators, highlighting the importance of experimental design in capturing these nuanced effects. This echoes the findings of prior studies suggesting that RF impacts depend on the quality and duration of exposure^18,28^. Despite counterarguments favouring single-factor models that emphasize temperature as the sole determinant of mosquito development, this study highlights the value of examining interaction effects. RF exposure, while secondary, holds potential as a modulator in mosquito biology. Further exploration of these combined effects can enhance our understanding of mosquito development under varying environmental and anthropogenic pressures, offering insights for more effective vector control strategies in the future. This study highlights the dominant role of temperature over RF exposure in influencing the developmental stages of *Ae. aegypti* and *Ae. albopictus*, with species-specific variations in sensitivity. The findings build on previous research by emphasizing the critical role of temperature in determining developmental success while suggesting that RF exposure may modulate these effects under certain conditions. These insights contribute to a growing understanding of environmental and anthropogenic factors influencing mosquito populations and provide a foundation for exploring innovative vector control strategies.

## Conclusion

In summary, temperature is the primary determinant of mosquito developmental durations, with RF exposure exerting secondary modulating effects under specific conditions. *Ae. aegypti* was more sensitive to environmental variations, while *Ae. albopictus* displayed greater resilience and adaptability. These findings provide a foundation for incorporating environmental variables, including anthropogenic factors such as RF exposure, into predictive models for mosquito population dynamics and vector management. Future research should explore the molecular mechanisms underlying these interactions and assess their implications for disease transmission and control in different ecological settings.

## Data Availability

All data generated or analyzed during this study are fully available without restriction in this manuscript and from the corresponding author.
